# Supplementary material for: A new natural killer cell-specific gene signature predicting recurrence in colorectal cancer patients
Source: Front Immunol. 2023 Jan 6;13:1011247. doi: 10.3389/fimmu.2022.1011247 (PMC9853446; doi:10.3389/fimmu.2022.1011247)
Supplement: Supplementary file 1 [file DataSheet_1.zip › UpdatedSupplementaryFiles.pdf]

Figure S1

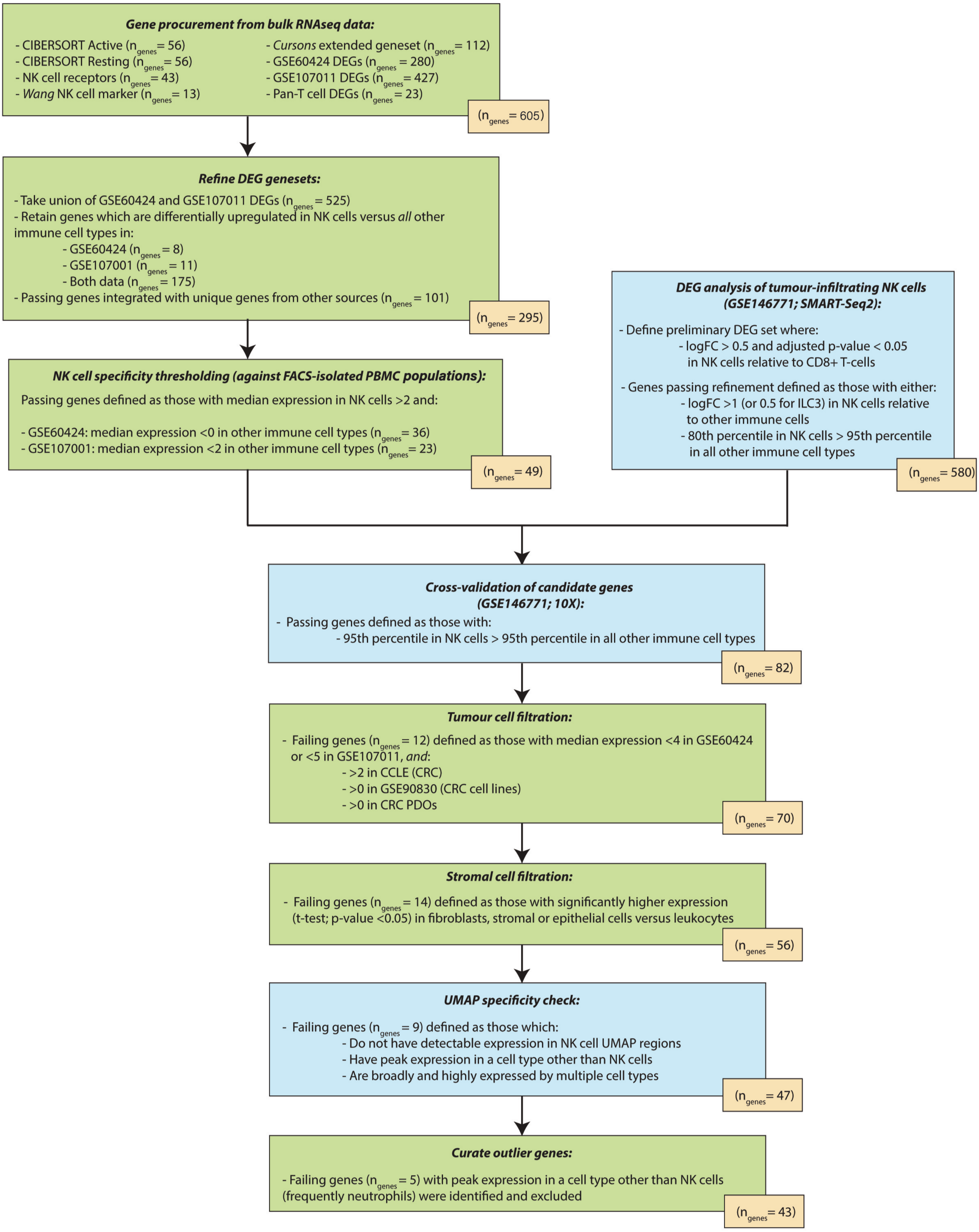
Gene procurement from bulk RNAseq data:
- CIBERSORT Active (n_{genes} = 56)
- CIBERSORT Resting (n_{genes} = 56)
- NK cell receptors (n_{genes} = 43)
- Wang NK cell marker (n_{genes} = 13)
- Cursons extended geneset (n_{genes} = 112)
- GSE60424 DEGs (n_{genes} = 280)
- GSE107011 DEGs (n_{genes} = 427)
- Pan-T cell DEGs (n_{genes} = 23)
(n_{genes} = 605)
Refine DEG genesets:
- Take union of GSE60424 and GSE107011 DEGs (n_{genes} = 525)
- Retain genes which are differentially upregulated in NK cells versus all other immune cell types in:
- GSE60424 (n_{genes} = 8)
- GSE107001 (n_{genes} = 11)
- Both data (n_{genes} = 175)
- Passing genes integrated with unique genes from other sources (n_{genes} = 101)
(n_{genes} = 295)
DEG analysis of tumour-infiltrating NK cells (GSE146771; SMART-Seq2):
- Define preliminary DEG set where:
- logFC > 0.5 and adjusted p-value < 0.05 in NK cells relative to CD8+ T-cells
- Genes passing refinement defined as those with either:
- logFC >1 (or 0.5 for ILC3) in NK cells relative to other immune cells
- 80th percentile in NK cells > 95th percentile in all other immune cell types
(n_{genes} = 580)
NK cell specificity thresholding (against FACS-isolated PBMC populations):
Passing genes defined as those with median expression in NK cells >2 and:
- GSE60424: median expression <0 in other immune cell types (n_{genes} = 36)
- GSE107001: median expression <2 in other immune cell types (n_{genes} = 23)
(n_{genes} = 49)
Cross-validation of candidate genes (GSE146771; 10X):
- Passing genes defined as those with:
- 95th percentile in NK cells > 95th percentile in all other immune cell types
(n_{genes} = 82)
Tumour cell filtration:
- Failing genes (n_{genes} = 12) defined as those with median expression <4 in GSE60424 or <5 in GSE107011, and:
- >2 in CCLE (CRC)
- >0 in GSE90830 (CRC cell lines)
- >0 in CRC PDOs
(n_{genes} = 70)
Stromal cell filtration:
- Failing genes (n_{genes} = 14) defined as those with significantly higher expression (t-test; p-value <0.05) in fibroblasts, stromal or epithelial cells versus leukocytes
(n_{genes} = 56)
UMAP specificity check:
- Failing genes (n_{genes} = 9) defined as those which:
- Do not have detectable expression in NK cell UMAP regions
- Have peak expression in a cell type other than NK cells
- Are broadly and highly expressed by multiple cell types
(n_{genes} = 47)
Curate outlier genes:
- Failing genes (n_{genes} = 5) with peak expression in a cell type other than NK cells (frequently neutrophils) were identified and excluded
(n_{genes} = 43)

Figure S2

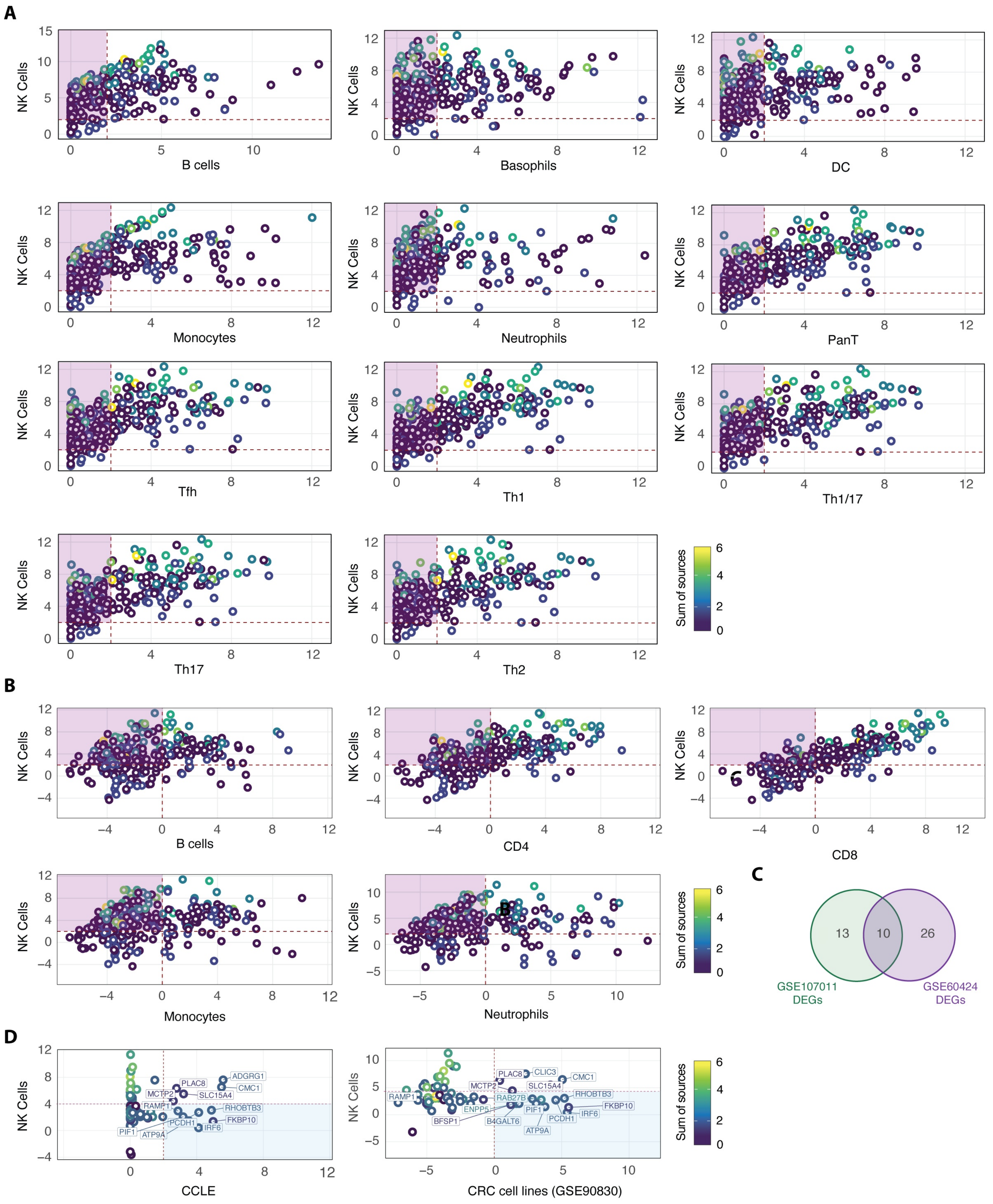
A
B
C
D
NK Cells
B cells
Basophils
DC
Monocytes
Neutrophils
PanT
Tfh
Th1
Th1/17
Th17
Th2
CD4
CD8
Sum of sources
GSE107011 DEGs
GSE60424 DEGs
13
10
26
CCLE
CRC cell lines (GSE90830)
ADGRG1
PLAC8
CMC1
MCTP2
SLC15A4
RAMP1
RHOBTB3
PCDH1
FKBP10
IRF6
PIF1
ATP9A
CLIC3
RAB27B
ENPP5
BFSP1
B4GALT6

Figure S3

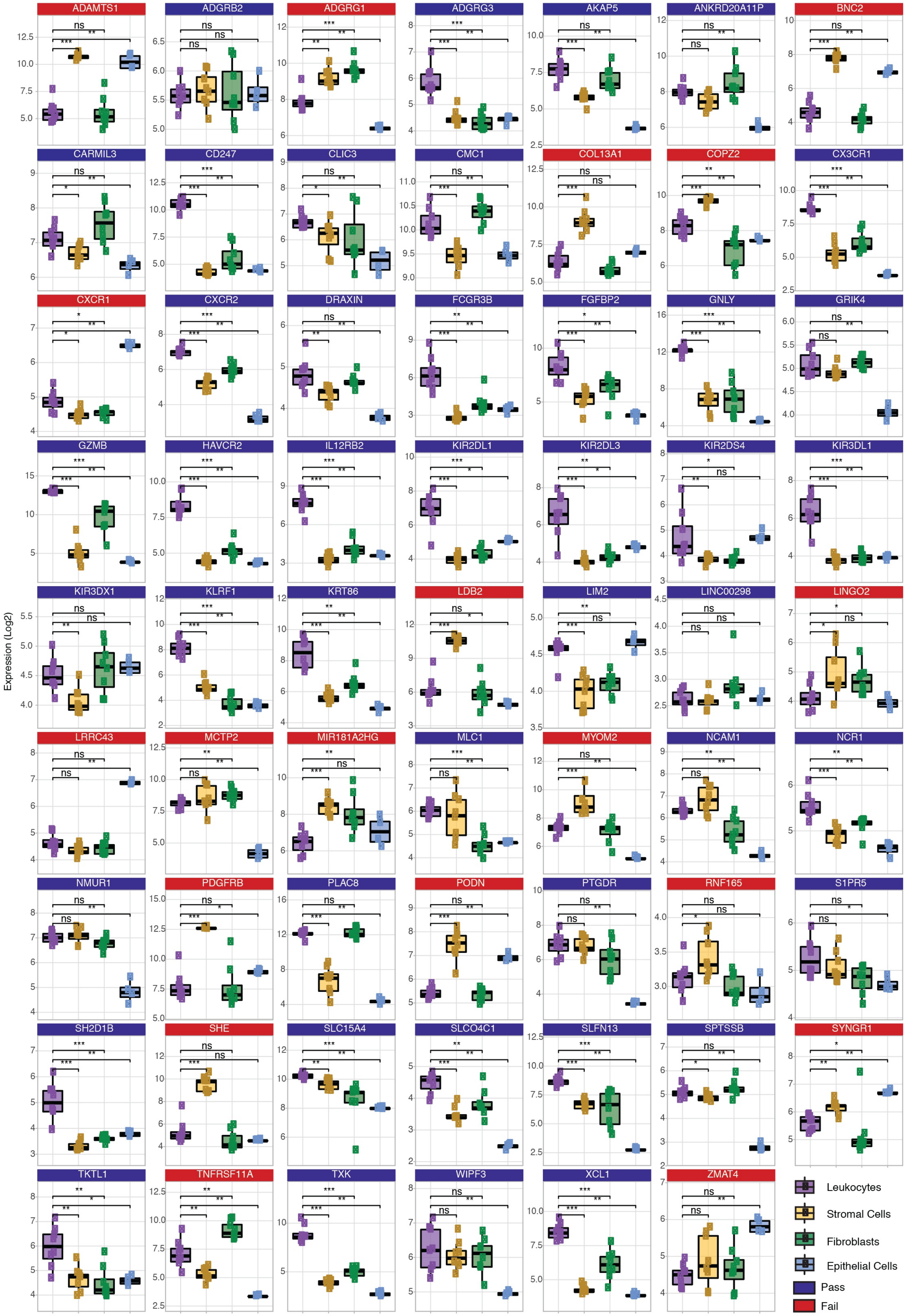
ADAMTS1
ADGRB2
ADGRG1
ADGRG3
AKAP5
ANKRD20A11P
BNC2
CARMIL3
CD247
CLIC3
CMC1
COL13A1
COPZ2
CX3CR1
CXCR1
CXCR2
DRAXIN
FCGR3B
FGFBP2
GNLY
GRIK4
GZMB
HAVCR2
IL12RB2
KIR2DL1
KIR2DL3
KIR2DS4
KIR3DL1
KIR3DX1
KLRF1
KRT86
LDB2
LIM2
LINC00298
LINGO2
LRRC43
MCTP2
MIR181A2HG
MLC1
MYOM2
NCAM1
NCR1
NMUR1
PDGFRB
PLAC8
PODN
PTGDR
RNF165
S1PR5
SH2D1B
SHE
SLC15A4
SLCO4C1
SLFN13
SPTSSB
SYNGR1
TKTL1
TNFRSF11A
TXK
WIPF3
XCL1
ZMAT4
Expression (Log2)
Leukocytes
Stromal Cells
Fibroblasts
Epithelial Cells
Pass
Fail

Figure S4

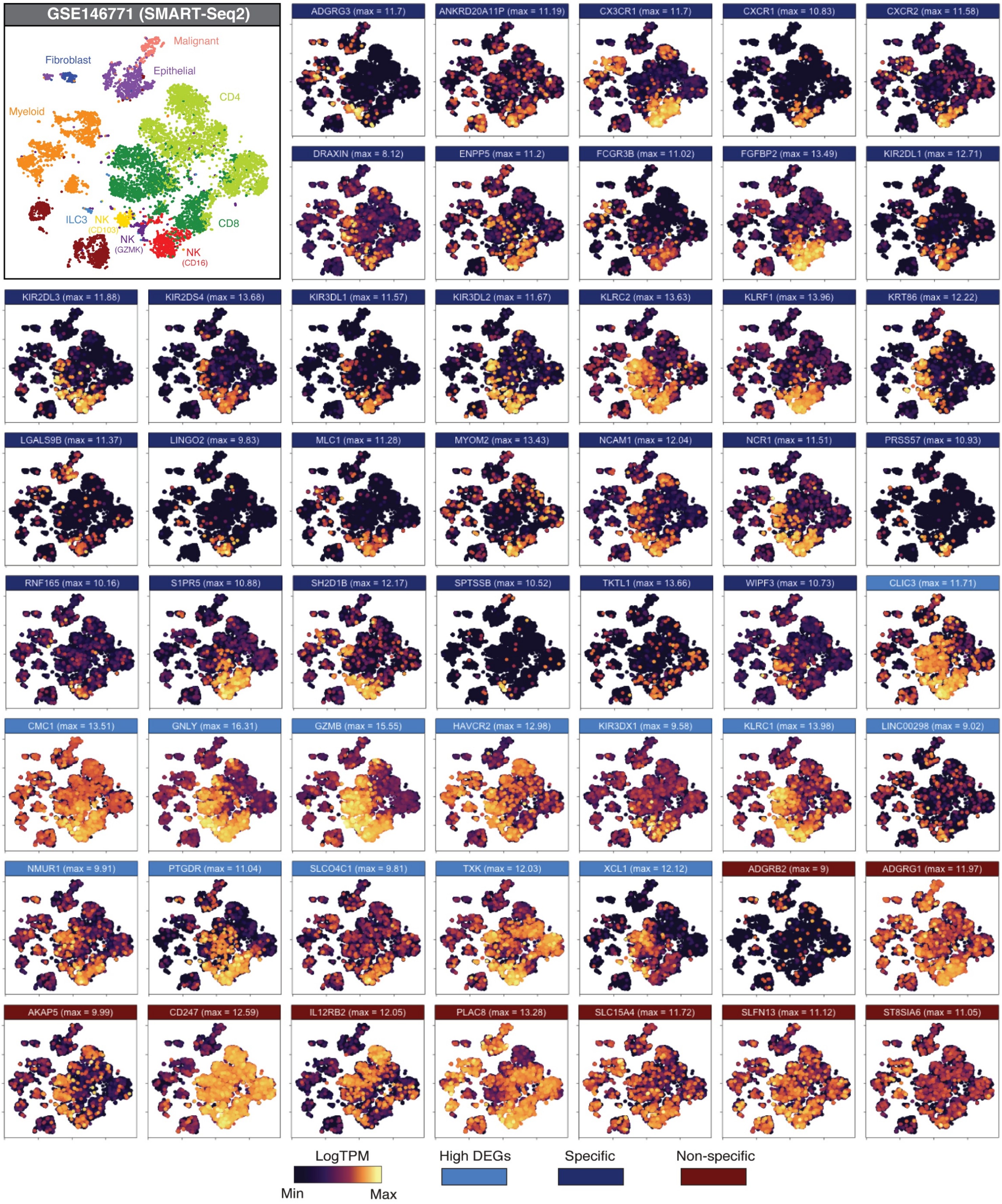
GSE146771 (SMART-Seq2)
Malignant
Fibroblast
Epithelial
CD4
Myeloid
ILC3
NK (CD103)
NK (GZMK)
CD8
NK (CD16)
ADGRG3 (max = 11.7)
ANKRD20A11P (max = 11.19)
CX3CR1 (max = 11.7)
CXCR1 (max = 10.83)
CXCR2 (max = 11.58)
DRAXIN (max = 8.12)
ENPP5 (max = 11.2)
FCGR3B (max = 11.02)
FGFBP2 (max = 13.49)
KIR2DL1 (max = 12.71)
KIR2DL3 (max = 11.88)
KIR2DS4 (max = 13.68)
KIR3DL1 (max = 11.57)
KIR3DL2 (max = 11.67)
KLRC2 (max = 13.63)
KLRF1 (max = 13.96)
KRT86 (max = 12.22)
LGALS9B (max = 11.37)
LINGO2 (max = 9.83)
MLC1 (max = 11.28)
MYOM2 (max = 13.43)
NCAM1 (max = 12.04)
NCR1 (max = 11.51)
PRSS57 (max = 10.93)
RNF165 (max = 10.16)
S1PR5 (max = 10.88)
SH2D1B (max = 12.17)
SPTSSB (max = 10.52)
TKTL1 (max = 13.66)
WIPF3 (max = 10.73)
CLIC3 (max = 11.71)
CMC1 (max = 13.51)
GNLY (max = 16.31)
GZMB (max = 15.55)
HAVCR2 (max = 12.98)
KIR3DX1 (max = 9.58)
KLRC1 (max = 13.98)
LINC00298 (max = 9.02)
NMUR1 (max = 9.91)
PTGDR (max = 11.04)
SLCO4C1 (max = 9.81)
TXK (max = 12.03)
XCL1 (max = 12.12)
ADGRB2 (max = 9)
ADGRG1 (max = 11.97)
AKAP5 (max = 9.99)
CD247 (max = 12.59)
IL12RB2 (max = 12.05)
PLAC8 (max = 13.28)
SLC15A4 (max = 11.72)
SLFN13 (max = 11.12)
ST8SIA6 (max = 11.05)
LogTPM
Min
Max
High DEGs
Specific
Non-specific

Figure S5

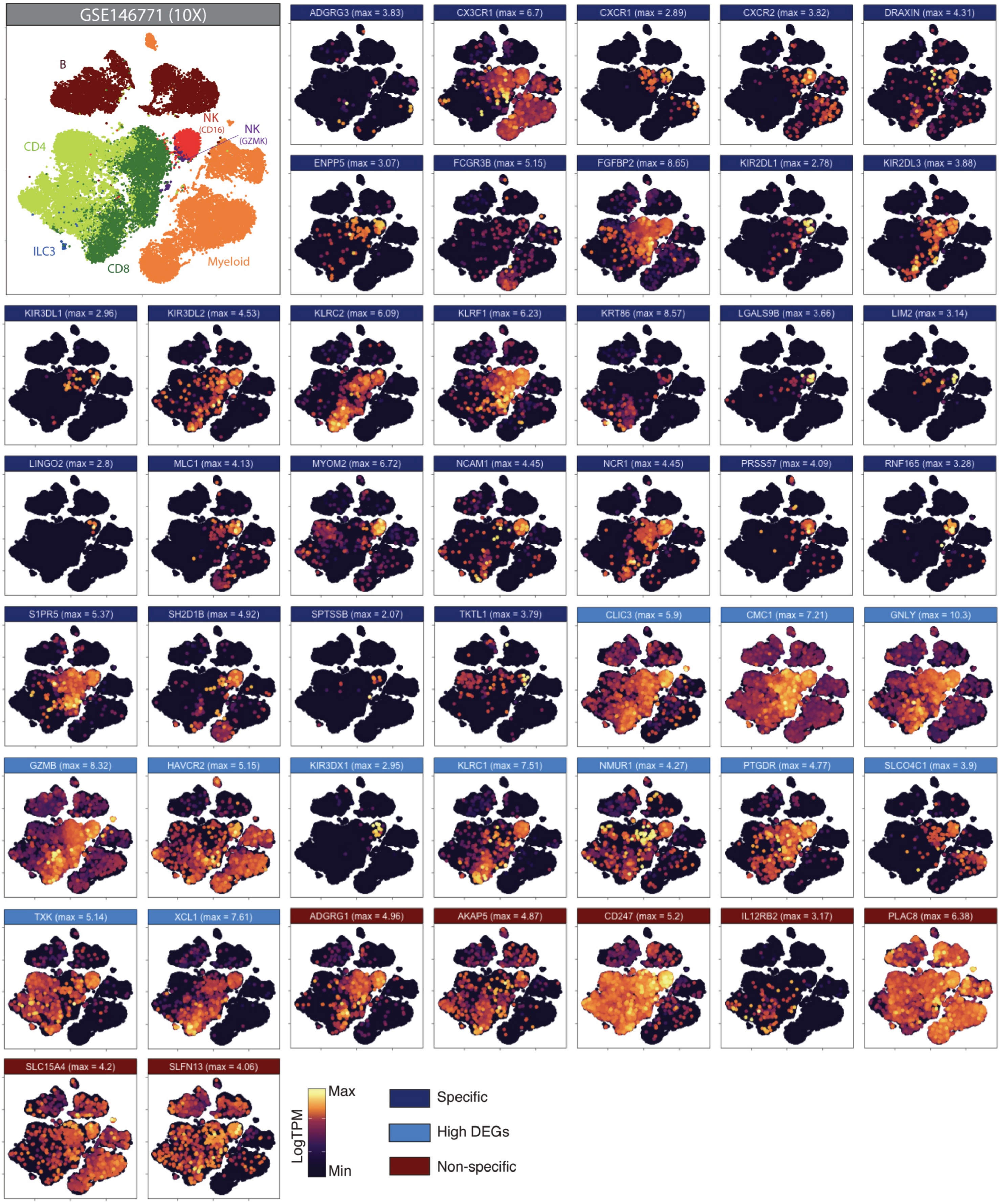

GSE146771 (10X)
B
NK (CD16)
NK (GZMK)
CD4
ILC3
CD8
Myeloid
ADGRG3 (max = 3.83)
CX3CR1 (max = 6.7)
CXCR1 (max = 2.89)
CXCR2 (max = 3.82)
DRAXIN (max = 4.31)
ENPP5 (max = 3.07)
FCGR3B (max = 5.15)
FGFBP2 (max = 8.65)
KIR2DL1 (max = 2.78)
KIR2DL3 (max = 3.88)
KIR3DL1 (max = 2.96)
KIR3DL2 (max = 4.53)
KLRC2 (max = 6.09)
KLRF1 (max = 6.23)
KRT86 (max = 8.57)
LGALS9B (max = 3.66)
LIM2 (max = 3.14)
LINGO2 (max = 2.8)
MLC1 (max = 4.13)
MYOM2 (max = 6.72)
NCAM1 (max = 4.45)
NCR1 (max = 4.45)
PRSS57 (max = 4.09)
RNF165 (max = 3.28)
S1PR5 (max = 5.37)
SH2D1B (max = 4.92)
SPTSSB (max = 2.07)
TKTL1 (max = 3.79)
CLIC3 (max = 5.9)
CMC1 (max = 7.21)
GNLY (max = 10.3)
GZMB (max = 8.32)
HAVCR2 (max = 5.15)
KIR3DX1 (max = 2.95)
KLRC1 (max = 7.51)
NMUR1 (max = 4.27)
PTGDR (max = 4.77)
SLCO4C1 (max = 3.9)
TXK (max = 5.14)
XCL1 (max = 7.61)
ADGRG1 (max = 4.96)
AKAP5 (max = 4.87)
CD247 (max = 5.2)
IL12RB2 (max = 3.17)
PLAC8 (max = 6.38)
SLC15A4 (max = 4.2)
SLFN13 (max = 4.06)
LogTPM
Max
Min
Specific
High DEGs
Non-specific

Figure S6

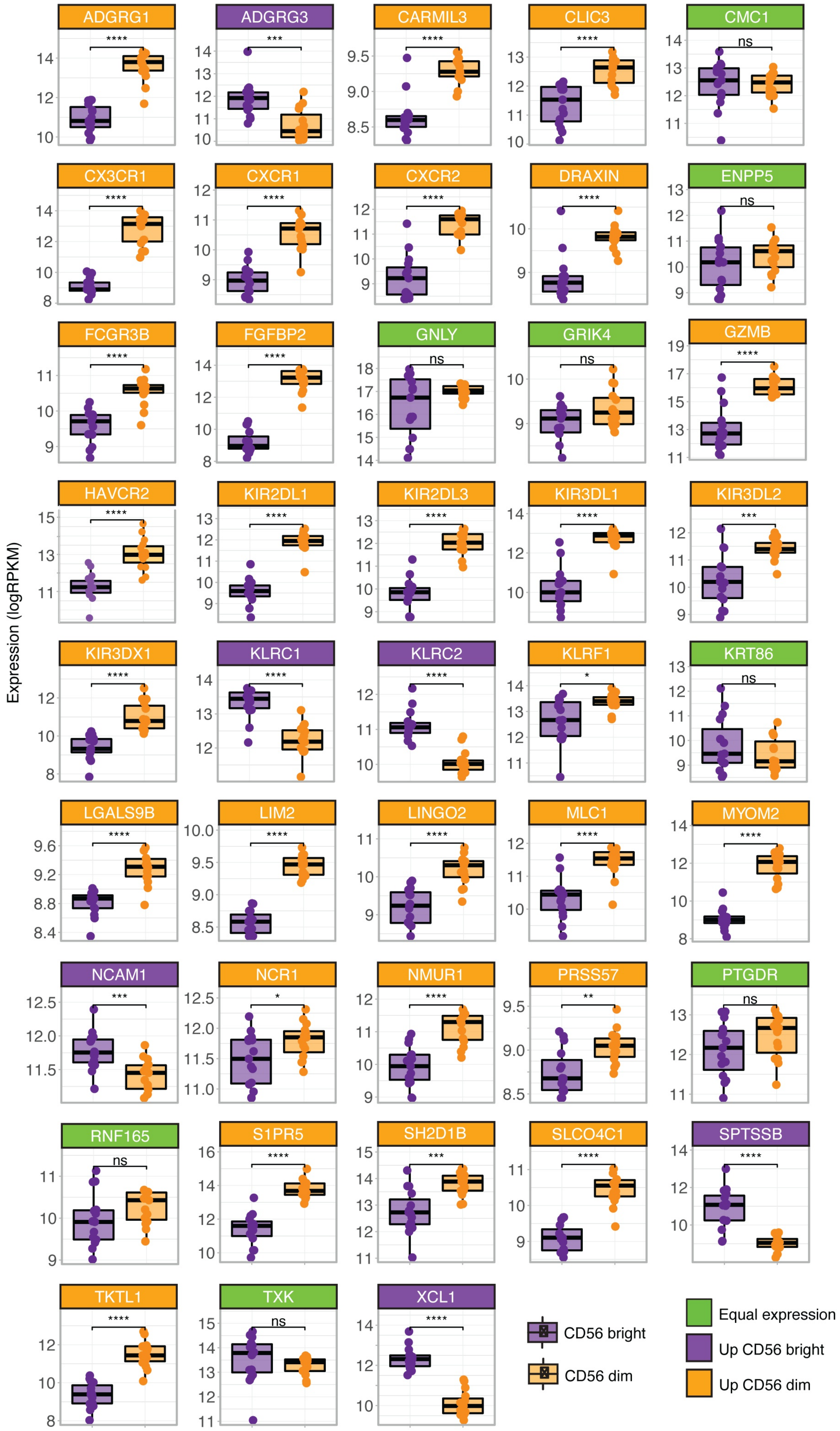
Expression (logRPKM)
ADGRG1 ****
ADGRG3 ***
CARMIL3 ****
CLIC3 ****
CMC1 ns
CX3CR1 ****
CXCR1 ****
CXCR2 ****
DRAXIN ****
ENPP5 ns
FCGR3B ****
FGFBP2 ****
GNLY ns
GRIK4 ns
GZMB ****
HAVCR2 ****
KIR2DL1 ****
KIR2DL3 ****
KIR3DL1 ****
KIR3DL2 ***
KIR3DX1 ****
KLRC1 ****
KLRC2 ****
KLRF1 *
KRT86 ns
LGALS9B ****
LIM2 ****
LINGO2 ****
MLC1 ****
MYOM2 ****
NCAM1 ***
NCR1 *
NMUR1 ****
PRSS57 **
PTGDR ns
RNF165 ns
S1PR5 ****
SH2D1B ***
SLCO4C1 ****
SPTSSB ****
TKTL1 ****
TXK ns
XCL1 ****
CD56 bright
CD56 dim
Equal expression
Up CD56 bright
Up CD56 dim

Figure S7

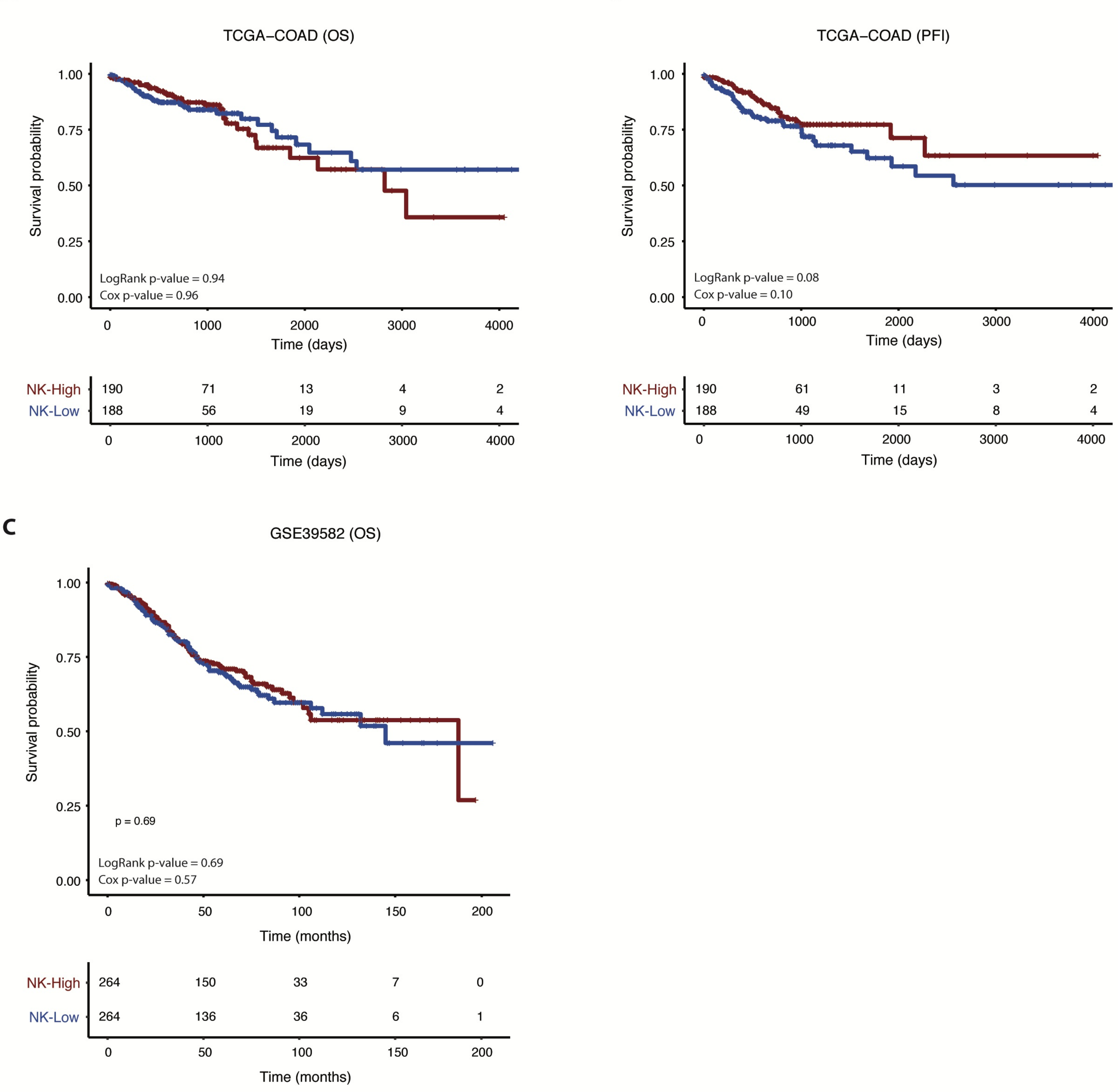
A
TCGA–COAD (OS)
Survival probability
1.00
0.75
0.50
0.25
0.00
LogRank p-value = 0.94
Cox p-value = 0.96
0
1000
2000
3000
4000
Time (days)
NK-High 190 71 13 4 2
NK-Low 188 56 19 9 4
B
TCGA–COAD (PFI)
LogRank p-value = 0.08
Cox p-value = 0.10
NK-High 190 61 11 3 2
NK-Low 188 49 15 8 4
C
GSE39582 (OS)
p = 0.69
LogRank p-value = 0.69
Cox p-value = 0.57
0
50
100
150
200
Time (months)
NK-High 264 150 33 7 0
NK-Low 264 136 36 6 1

Figure S8

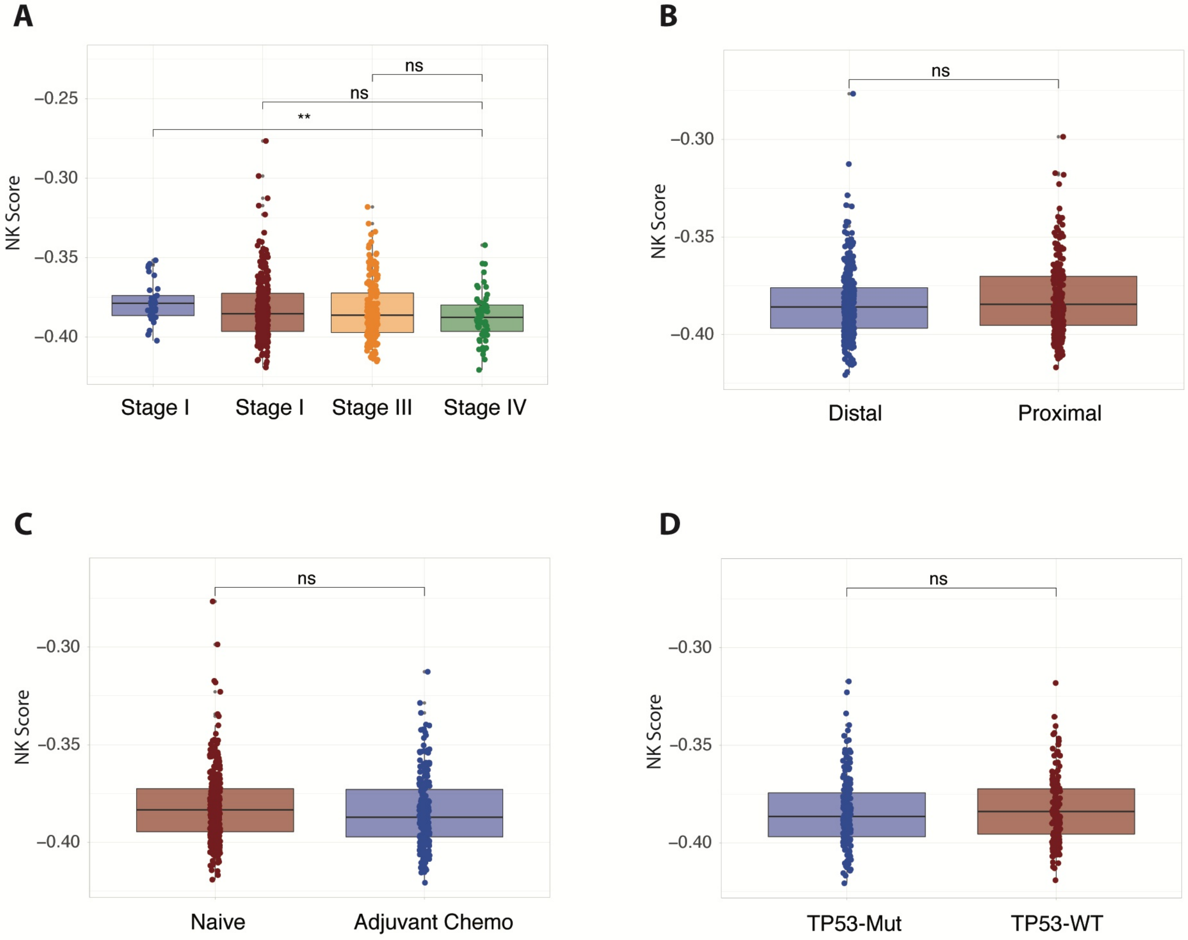
A
NK Score
−0.25
−0.30
−0.35
−0.40
ns
ns
**
Stage I
Stage I
Stage III
Stage IV
B
NK Score
−0.30
−0.35
−0.40
ns
Distal
Proximal
C
NK Score
−0.30
−0.35
−0.40
ns
Naive
Adjuvant Chemo
D
NK Score
−0.30
−0.35
−0.40
ns
TP53-Mut
TP53-WT

Figure S9

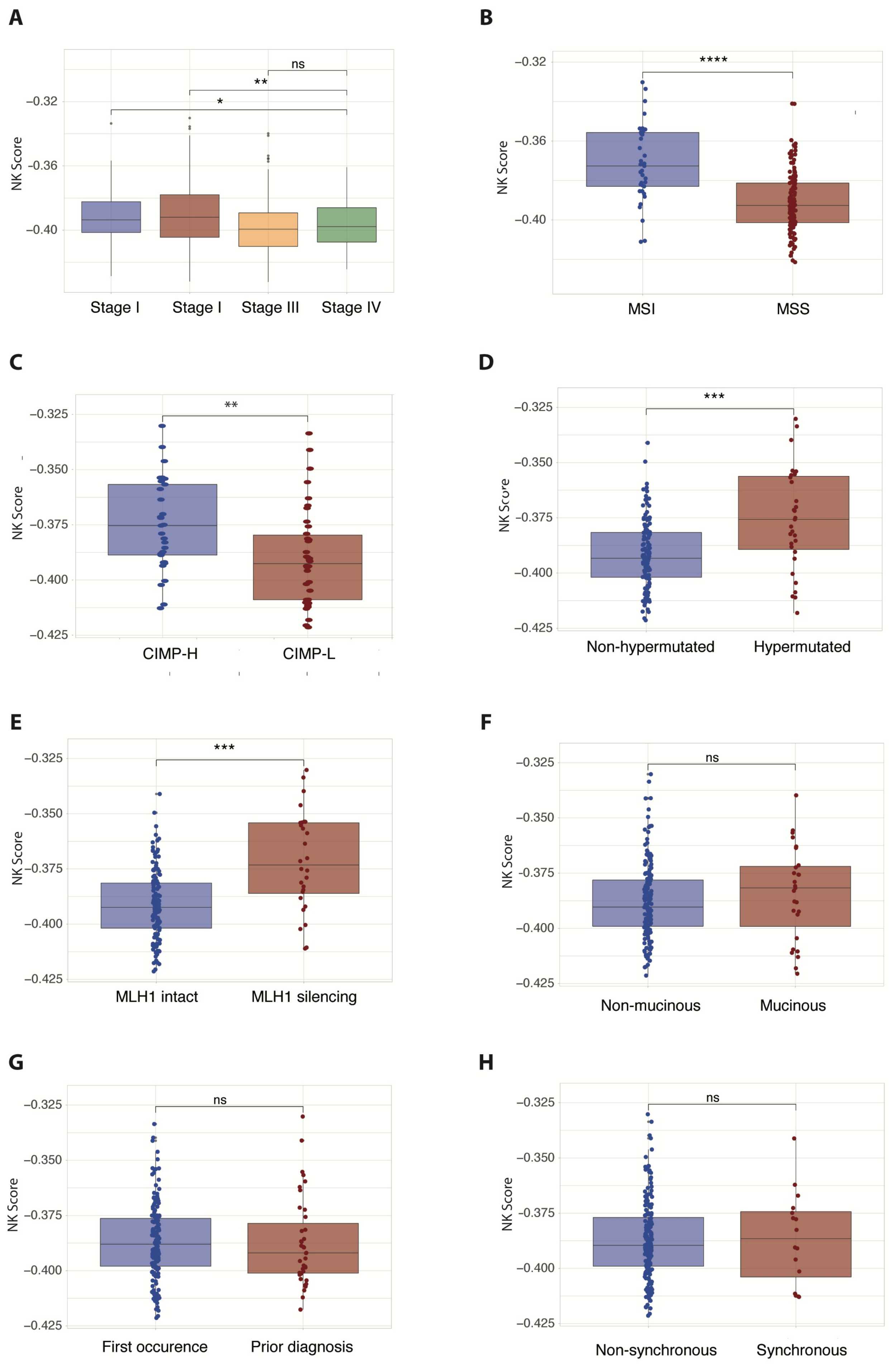
A
NK Score
−0.32
−0.36
−0.40
ns
**
*
Stage I
Stage I
Stage III
Stage IV
B
NK Score
−0.32
−0.36
−0.40
****
MSI
MSS
C
NK Score
−0.325
−0.350
−0.375
−0.400
−0.425
**
CIMP-H
CIMP-L
D
NK Score
−0.325
−0.350
−0.375
−0.400
−0.425
***
Non-hypermutated
Hypermutated
E
NK Score
−0.325
−0.350
−0.375
−0.400
−0.425
***
MLH1 intact
MLH1 silencing
F
NK Score
−0.325
−0.350
−0.375
−0.400
−0.425
ns
Non-mucinous
Mucinous
G
NK Score
−0.325
−0.350
−0.375
−0.400
−0.425
ns
First occurence
Prior diagnosis
H
NK Score
−0.325
−0.350
−0.375
−0.400
−0.425
ns
Non-synchronous
Synchronous
